# Autoimmune GAD-positive rhombencephalitis with rare manifestation: a case report and literature review

**DOI:** 10.3389/fmed.2025.1721582

**Published:** 2026-01-20

**Authors:** Anas Khaled Zurayqi, Anas R. Tuqan, Anas M. Barabrah, Basel A. Zaben, Ruba Saadeh

**Affiliations:** Faculty of Medicine, Al-Quds University, Jerusalem, Palestine

**Keywords:** autoimmune, case report, glutamic acid decarboxylase (GAD) antibody, hydrophobia, rhombencephalitis

## Abstract

GAD-positive rhombencephalitis is a rare autoimmune neurological disorder characterized by the presence of antibodies targeting glutamic acid decarboxylase (GAD), an enzyme essential for neurotransmitter regulation. This case report presents a 43-year-old male patient with progressive neurological symptoms, including gait instability, cranial nerve palsy, dysphagia, dysarthria, and hydrophobia. Diagnostic evaluation showed a modest elevation in serum anti-GAD antibodies, while brain MRI demonstrated mild cerebellar atrophy. Electroencephalography (EEG) revealed multifocal slowing and sharp wave activity in the bitemporal and central regions, consistent with diffuse cerebral dysfunction without epileptiform discharges. In addition, an extensive workup was performed to exclude infectious, metabolic, paraneoplastic, and alternative autoimmune etiologies. Initial treatment with pulse corticosteroids and intravenous immunoglobulin (IVIG) provided partial symptom relief; however, the patient subsequently experienced respiratory distress and pneumonia, requiring intensive care intervention. The clinical presentation in this case exemplifies the diagnostic intricacy of anti-GAD antibody-related disorders, which frequently exhibit symptoms that overlap with other autoimmune and infectious encephalitis. This case illustrates the value of identifying unique manifestations such as hydrophobia, which has not been previously reported in the context of anti-GAD rhombencephalitis. Treatment focused on immunotherapy, including high-dose corticosteroids and plasmapheresis, while emphasis was placed on vigilant management of potential complications and concurrent autoimmune conditions. This report contributes to the understanding of anti-GAD antibody-related neurological syndromes and highlights the importance of a multidisciplinary approach in managing these complex cases.

## Introduction

Autoimmune GAD-positive rhombencephalitis is an uncommon yet clinically significant neurological disorder marked by the presence of antibodies targeting glutamic acid decarboxylase (GAD), an enzyme crucial for neurotransmitter regulation in the central nervous system (1). Neurological syndromes associated with anti-GAD antibodies are uncommon, with an estimated prevalence of 1.9 per 100,000 for limbic encephalitis (LE) (2). This autoimmune disorder is marked by diverse clinical presentations, including seizures, cognitive deterioration, and movement disorders, and it may be accompanied by secondary autoimmune conditions, such as type 1 diabetes mellitus and autoimmune thyroiditis (3–5). The underlying pathophysiology involves an aberrant immune response targeting neuronal tissues, culminating in inflammation and functional impairment within specific brain regions.

The clinical relevance of anti-GAD antibody positivity is significant, as prompt identification and treatment are key to maximizing patient outcomes. Immunotherapies, such as corticosteroids and intravenous immunoglobulin (IVIG), have demonstrated effectiveness in mitigating symptoms and improving quality of life (5, 6). Nonetheless, considerable challenges remain in delineating the full spectrum of anti-GAD antibody-related neurological manifestations and elucidating the pathophysiological mechanisms of this immune-mediated process.

This case report seeks to illustrate the clinical presentation, diagnostic obstacles, and therapeutic management of a patient diagnosed with GAD-positive rhombencephalitis, further complicated by hydrophobia.

This case has been reported in line with the CARE criteria (7).

## Case presentation

A 43-year-old male computer engineer was referred to our neurology service in March 2024 for a trial of therapeutic plasma exchange. His initial symptoms began 18 months earlier with chronic mid-back pain associated with difficulty in walking, attributable to mechanical discomfort rather than weakness, rigidity, or sensory loss. His condition remained stable until December 2023, when he began experiencing new symptoms, such as gait unsteadiness, episodic diplopia, imbalance, dysarthria, and dysphagia.

In December 2023, the patient was treated with high-dose pulse corticosteroid therapy, which resulted in partial clinical improvement; however, symptoms recurred and progressed. On 11 February 2024, he subsequently received a 5-day course of intravenous immunoglobulin (IVIG) at a dose of 0.48 g/kg/day, producing only a transient benefit. Continued clinical deterioration prompted referral to our service on 6 March 2024. Serum anti-glutamic acid decarboxylase (GAD) antibodies were obtained on 22 February 2024, approximately 2 months after completion of corticosteroid therapy and 11 days after initiation of IVIG, a timing that may have influenced measured antibody titers.

On admission, the patient exhibited significant clinical deterioration. He was unable to ambulate independently due to severe cerebellar ataxia, without evidence of limb weakness, spasticity, or rigidity. He exhibited severe mixed dysarthria, which included both cerebellar and bulbar features, characterized by difficulty with articulation due to muscle weakness and a scanning, ataxic speech pattern. He reported choking on both solids and liquids. He denied sensory symptoms, limb weakness, and bladder dysfunction but noted constipation. He also developed intermittent agitation and depressive symptoms. Over the preceding months, his family observed an unusual behavioral change: hydrophobic tendencies characterized by fear of running water, refusal to shower, and difficulty drinking unless fluids were provided in dark, opaque containers. Rabies exposure was conclusively excluded, and a psychiatric etiology was not supported clinically.

The patient was alert, oriented, and cognitively intact, exhibiting normal speech content but severe mixed dysarthria. The examination demonstrated tongue fasciculations, cranial nerve IV and VI palsies, and absent elevation of the palate and uvula, although the gag reflex remained intact. Muscle bulk, tone, and strength were normal (5/5 in all extremities). Deep tendon reflexes were diffusely brisk, with spreading bilateral cross-adductor responses, extensor plantar responses, and non-sustained clonus. The sensory examination was normal. Cerebellar testing revealed marked truncal and limb ataxia, impaired finger-to-nose coordination, and dysdiadochokinesia. The ocular examination demonstrated abnormal saccadic gaze and non-sustained horizontal nystagmus.

Of note, and relevant to the differential diagnosis of progressive encephalomyelitis with rigidity and myoclonus (PERM), the patient exhibited normal muscle tone with no axial or limb rigidity, no stimulus-sensitive spasms, no exaggerated startle response, no myoclonus, and no episodes of laryngospasm.

Serum anti-GAD antibody level was 39.40 IU/mL (reference < 5 IU/mL), obtained via quantitative ELISA. Given the timing—weeks after corticosteroids and IVIG—the result may not reflect the true baseline titer due to potential transient suppression or interference. CSF anti-GAD testing was not available, limiting assessment of intrathecal synthesis. A comprehensive autoimmune encephalitis panel (anti-NMDAR, AMPAR, LGI1, Caspr2, GABA-B, mGluR1/5, GlyR, DPPX) was negative. Screening for Wilson’s disease was unremarkable.

Brain and cervical spine MRI demonstrated mild cerebellar atrophy without acute lesions. EEG showed multifocal slow and sharp wave activity predominantly in the bitemporal and central regions, indicating diffuse cerebral dysfunction.

Given the subacute progression, the presence of objective brainstem and cerebellar deficits, MRI findings of mild cerebellar atrophy, EEG evidence of diffuse encephalopathy, and the unusual hydrophobic behaviors—together with a moderately elevated anti-GAD antibody level interpreted cautiously in the context of recent immunotherapies and the exclusion of alternative causes—we diagnosed the patient with autoimmune GAD-positive rhombencephalitis.

The patient underwent three sessions of therapeutic plasma exchange, which were well tolerated and led to partial improvement in gait stability. One day after the third session, he developed acute respiratory compromise associated with *Haemophilus influenzae* pneumonia, confirmed by sputum and blood cultures. CT pulmonary angiography and echocardiography ruled out pulmonary embolism. He required prolonged mechanical ventilation. On day 7 of intubation, sedation was reduced, revealing appropriate command following, intact cranial motor responses, and spontaneous limb movements. Sedation was resumed due to tachypnea, and he was eventually extubated on day 56 of admission. He received rituximab 1 g on days 0 and 14, initiated 6 weeks after admission.

At discharge, the patient was alert, oriented, and neurologically stable, exhibiting persistent but non-progressive cerebellar ataxia and abnormal dysarthria. Although no objective neurological recovery was noted, his condition had stabilized without further deterioration, and hydrophobic behaviors remained unchanged.

At a 4-month follow-up, he remained clinically stable with no further neurological deterioration. However, there was no evidence of improvement, and he still reported hydrophobia.

## Discussion

Glutamic acid decarboxylase (GAD) is an intracellular enzyme predominantly expressed in neurons and insulin-producing pancreatic *β* cells, where it facilitates the conversion of glutamate into gamma-aminobutyric acid (GABA) (8). Neurological syndromes associated with GAD autoimmunity are relatively rare. GAD antibodies (Ab) account for about 2% of sporadic progressive cerebellar ataxia (CA) (9) and 12% of CA of unknown origin (10), indicating their significance in cerebellar dysfunction. In limbic encephalitis (LE), GAD Ab is detected in roughly 17% of patients (11). These neurological conditions exhibit a marked female predominance, with over 80% of cases occurring in women (12, 13). The median age of onset for LE typically ranges from 25 to 45 years (11, 12).

Patients with GAD Ab-related neurological disorders often have personal or familial histories of autoimmune diseases. A family history of autoimmune conditions, such as type 1 diabetes mellitus (T1DM) or thyroiditis, is prevalent, seen in 43–55% of patients with stiff-person syndrome (SPS) and CA and in 23% of those with temporal lobe epilepsy (TLE) (14, 15). Approximately half of these patients report a personal history of T1DM, frequently preceding the onset of neurological symptoms (1). In addition to T1DM, many individuals exhibit other manifestations of systemic autoimmunity, such as thyroiditis, pernicious anemia, celiac disease, and vitiligo. The presence of various autoantibodies, such as anti-thyroid peroxidase and anti-gliadin antibodies, underscores the connection between GAD autoimmunity and other systemic autoimmune conditions. There is also a higher incidence of cancer associated with GAD autoimmunity in CA and LE, with paraneoplastic cases reported at 9 and 26%, respectively (16).

GABAergic neurons form a crucial network of inhibitory interneurons throughout the central nervous system, primarily located in the hippocampus, cerebellum, basal ganglia, brainstem nuclei, and spinal gray matter (17). These neurons produce high levels of GAD65, the enzyme responsible for synthesizing GABA. Following its synthesis in presynaptic terminals, GABA is stored in synaptic vesicles and released into the synaptic cleft via exocytosis (18). Once released, GABA binds to GABAA and GABAB receptors, resulting in the hyperpolarization of postsynaptic neurons and the generation of inhibitory signals (19). Although the precise mechanisms by which GAD autoimmunity affects GABAergic transmission are not fully understood, it is clear that GAD Ab disrupts this process. It is hypothesized that GAD Ab inhibits GAD65, thereby blocking GABA synthesis by reducing the uptake of newly synthesized GABA into synaptic vesicles and its subsequent release (1, 18, 20). This reduction in GABA synthesis subsequently leads to diminished GABAergic transmission and a state of neuronal hyperexcitability (21). *In vitro* studies have shown that GAD Ab from CA patients decreases presynaptic GABA release, particularly in Purkinje cells, contributing to CA pathogenesis (1). While data on GABAergic transmission alterations in GAD-associated LE are limited, *in vivo* evidence suggests that reduced GABAergic transmission may lead to cortical hyperexcitability, as seen in GAD65 knockout mice that develop seizures in limbic regions (22).

The primary neurological conditions linked to GAD antibodies include stiff-person syndrome (SPS), cerebellar ataxia (CA), and, less frequently, limbic encephalitis (LE) (23, 24). SPS is characterized by axial rigidity, painful muscle spasms, and a tendency to fall, often triggered by unexpected stimuli or emotional stress (1). CA typically manifests with gait disturbances and cerebellar atrophy, while LE is marked by seizures and cognitive impairment. Overlap syndromes, where patients exhibit symptoms of multiple conditions, are observed in 10–20% of SPS cases, with similar percentages in CA and LE. In addition, GAD antibodies have been associated with other neurological manifestations, such as progressive encephalomyelitis with rigidity and myoclonus, opsoclonus-myoclonus, and autonomic neuropathy (1). Notably, our patient presented with hydrophobia, a symptom not previously documented in the literature.

The differential diagnosis of rhombencephalitis is broad and includes paraneoplastic, infectious, autoimmune, metabolic, and demyelinating conditions. Paraneoplastic rhombencephalitis, linked to cancers like small-cell lung carcinoma, shares features like cranial nerve dysfunction and ataxia, which were reasonably excluded by negative malignancy-specific antibodies (e.g., Hu, Ma2) and unique MRI patterns (25, 26). Infectious rhombencephalitis, often due to *Listeria monocytogenes*, presents with fever and focal deficits and shows asymmetric brainstem lesions on MRI, features absent in our patient (27). Other autoimmune encephalitis (e.g., anti-NMDA, VGKC-complex, and anti-AMPA) and conditions like Bickerstaff brainstem encephalitis, multiple sclerosis, Wilson’s disease, and metabolic disorders (e.g., Wernicke’s encephalopathy) were systematically excluded through neuroimaging, laboratory testing, and antibody panels (26, 27).

Patients with anti-GAD antibody-related autoimmune encephalitis often experience complex complications affecting their quality of life. Refractory seizures are common, challenging control with standard medications (3). Cognitive impairments such as memory loss and executive dysfunction are prevalent, which can hinder daily activities and independence (4). Mood disorders, including anxiety and depression, frequently arise, likely worsened by the chronic illness (3, 5). Patients may also develop secondary autoimmune conditions like type 1 diabetes and autoimmune thyroiditis, complicating their clinical management and requiring ongoing monitoring and treatment (4). Some may exhibit symptoms of stiff-person syndrome, marked by muscle stiffness and spasms, further impairing mobility and quality of life (3, 5). A multidisciplinary approach with neurologists, psychiatrists, and endocrinologists is essential to manage these multifaceted complications effectively.

The diagnosis of GAD-positive rhombencephalitis is based on recognizing a characteristic combination of clinical features, serological findings, and supportive neuroimaging. Patients often present with neurological symptoms such as cerebellar ataxia, cognitive dysfunction, or cranial nerve deficits, which may overlap with paraneoplastic or other autoimmune syndromes (25, 26). A distinguishing feature is the detection of high-titer anti-GAD antibodies in the serum or cerebrospinal fluid (CSF). These antibody titers, although supportive of the diagnosis, do not correlate directly with disease severity or treatment response (28, 29). Previous studies have reported the presence of both serum and CSF anti-GAD antibodies in approximately 60% of cases (5), further underscoring the diagnostic value of paired testing.

A critical challenge in interpreting GAD antibodies lies in their titer-dependent specificity and the influence of immunotherapy timing. Low-titer serum positivity is common in the general population, and ELISA-based assays may detect low levels in individuals with no neurological disease. Clinically significant associations generally manifest at exceedingly high serum titers, frequently surpassing 10,000 IU/mL, or in the presence of antibodies within the cerebrospinal fluid (CSF), indicative of intrathecal synthesis (29, 30). In our patient, the serum anti-GAD level was 39.4 IU/mL, measured 11 days after initiation of IVIG (administered for 5 days at a dose of 0.48 g/kg/day), which may have transiently suppressed antibody titers or caused assay interference.

Previous research has demonstrated that IVIG therapy can induce a delayed decline in anti-GAD titers through anti-idiotypic antibody mechanisms, with reductions observed within 1 week and nadir levels reached by approximately 3 weeks after treatment, in addition to broader immunomodulatory effects on T cells and cytokines (31). Accordingly, the measured antibody level in this case cannot be considered a definitive diagnostic marker, and interpretation must be cautious. CSF anti-GAD testing was unavailable, limiting confirmation of intrathecal synthesis. Therefore, the antibody result is regarded as supportive evidence in the context of the clinical presentation rather than a stand-alone diagnostic criterion.

Neuroimaging, particularly brain MRI, aids in diagnosis; although findings are often subtle or non-specific, mild cerebellar atrophy may be observed, reflecting possible chronicity. EEG studies frequently show generalized or multifocal slowing, which supports a diagnosis of diffuse encephalopathy but lacks the specificity required for a definitive diagnosis (28). MRI brain abnormalities are reported in approximately 78% of cases and EEG abnormalities in around 77% (5). In our patient, brain MRI revealed mild cerebellar atrophy, while EEG demonstrated multifocal slow and sharp wave activity, predominantly in the bitemporal and central regions, indicating diffuse cerebral dysfunction without epileptiform discharges.

Given the limited disease specificity associated with low-titer anti-glutamic acid decarboxylase (GAD) antibodies, the diagnostic evaluation was guided by the international criteria for possible autoimmune encephalitis proposed by Graus et al. (32). These criteria require a subacute (< 3 months) onset of altered mental status, psychiatric symptoms, or working memory deficits; the presence of at least one supportive feature—such as focal central nervous system deficits, seizures, or cerebrospinal fluid (CSF) or MRI abnormalities; and the reasonable exclusion of alternative etiologies (32).

The patient fulfilled these criteria for possible autoimmune encephalitis with the clinical phenotype. He developed a subacute neurological syndrome accompanied by prominent psychiatric manifestations, including agitation and depressive symptoms. Although there was no impairment of consciousness or objective working memory deficit, the presence of psychiatric symptoms satisfied the core clinical criterion. Furthermore, he exhibited new focal neurological deficits involving the cerebellum and brainstem. Electroencephalography demonstrated bitemporal multifocal slowing with sharp waves, consistent with diffuse cerebral dysfunction. Brain MRI revealed mild cerebellar atrophy. An extensive diagnostic workup, such as testing for NMDAR, AMPAR, LGI1, CASPR2, GABA-B receptor, glycine receptor, and paraneoplastic antibodies, failed to identify an alternative cause, and a partial response to immunotherapy collectively supports the diagnosis.

Nevertheless, diagnostic certainty is limited by the absence of CSF anti-GAD antibody testing. In addition, serum antibody measurement was performed after initiation of immunotherapy, which may have reduced antibody titers and further complicated interpretation.

The management of autoimmune GAD-positive rhombencephalitis emphasizes immunotherapy with high-dose corticosteroids as the primary intervention. Patients with severe or refractory symptoms, such as in our case, may receive additional therapies such as IV immunoglobulin (IVIG) or therapeutic plasma exchange (TPE), while long-term immunosuppressants like azathioprine or rituximab help prevent relapse. In cases complicated by seizures or significant neurological deficits, adjunctive anti-epileptic medications and intensive care support may be required (3, 6). A comprehensive, multidisciplinary approach combining immunotherapy with early and structured rehabilitation focused on balance and gait training is crucial for enhancing functional recovery and reducing long-term disability in patients with anti-GAD encephalitis. Psychological support for neuropsychiatric symptoms also contributes to overall recovery and quality of life, as does psychological support for the patient’s caregivers (33).

## Conclusion

This case report highlights the clinical and diagnostic challenges associated with GAD-positive rhombencephalitis, a rare autoimmune neurological condition with diverse manifestations. The patient presented with an uncommon symptom profile, particularly hydrophobia, which is rarely described in the literature and therefore expands current understanding of possible presentations in such cases. Diagnosis was supported through careful clinical correlation, supported by low-titer serum anti-GAD antibody positivity and compatible radiological and electrophysiological findings, acknowledging the potential impact of prior immunotherapy on antibody levels. Treatment involved corticosteroids, intravenous immunoglobulin, and therapeutic plasma exchange, reflecting the complexity of managing this condition. This case underscores the value of early recognition, careful diagnostic evaluation, and coordinated multidisciplinary care when dealing with autoimmune GAD-positive rhombencephalitis and its atypical presentations.

## Data Availability

The datasets presented in this study can be found in online repositories. The names of the repository/repositories and accession number(s) can be found in the article/supplementary material.
